# Signal variance-based collateral index in DSC perfusion: A novel method to assess leptomeningeal collateralization in acute ischaemic stroke

**DOI:** 10.1177/0271678X19831024

**Published:** 2019-02-13

**Authors:** Alexander Seiler, Arne Lauer, Ralf Deichmann, Ulrike Nöth, Eva Herrmann, Joachim Berkefeld, Oliver C Singer, Waltraud Pfeilschifter, Johannes C Klein, Marlies Wagner

**Affiliations:** 1 Department of Neurology, Goethe University Frankfurt, Frankfurt, Germany; 2 Institute of Neuroradiology, Goethe University Frankfurt, Frankfurt, Germany; 3 Brain Imaging Center, Goethe University Frankfurt, Frankfurt, Germany; 4 Institute of Biostatistics and Mathematical Modelling, Goethe University Frankfurt, Frankfurt, Germany; 5 Wellcome Centre for Integrative Neuroimaging, FMRIB, Nuffield Department of Clinical Neurosciences, University of Oxford, Oxford, UK; 6 Nuffield Department of Clinical Neurosciences, University of Oxford, Oxford, UK

**Keywords:** Acute ischaemic stroke, collaterals, functional outcome, magnetic resonance imaging, perfusion-weighted imaging

## Abstract

As a determinant of the progression rate of the ischaemic process in acute large-vessel stroke, the degree of collateralization is a strong predictor of the clinical outcome after reperfusion therapy and may influence clinical decision-making. Therefore, the assessment of leptomeningeal collateralization is of major importance. The purpose of this study was to develop and evaluate a quantitative and observer-independent method for assessing leptomeningeal collateralization in acute large-vessel stroke based on signal variance characteristics in T2*-weighted dynamic susceptibility contrast (DSC) perfusion-weighted MR imaging (PWI). Voxels representing leptomeningeal collateral vessels were extracted according to the magnitude of signal variance in the PWI raw data time series in 55 patients with proximal large-artery occlusion and an intra-individual collateral vessel index (CVI_PWI_) was calculated. CVI_PWI_ correlated significantly with the initial ischaemic core volume (rho = −0.459, p = 0.0001) and the PWI/DWI mismatch ratio (rho = 0.494, p = 0.0001) as an indicator of the amount of salvageable tissue. Furthermore, CVI_PWI_ was significantly negatively correlated with NIHSS and mRS at discharge (rho = −0.341, p = 0.015 and rho = −0.305, p = 0.023). In multivariate logistic regression, CVI_PWI_ was an independent predictor of favourable functional outcome (mRS 0–2) (OR = 16.39, 95% CI 1.42–188.7, p = 0.025). CVI_PWI_ provides useful rater-independent information on the leptomeningeal collateral supply in acute stroke.

## Introduction

Endovascular revascularization is highly effective in patients with acute ischaemic stroke due to proximal large-artery occlusion.^1^ Recently, the benefit of reperfusion has been confirmed, even for patients presenting beyond formerly established time windows but showing a present mismatch between the clinical deficit, respectively, the relevantly hypoperfused but salvageable tissue and the infarct volume.^2,3^ The latter strongly depends on the individual collateral capacity which is a determinant of both the amount of salvageable tissue and the progression rate of the ischaemic process as well as a predictor of the clinical outcome after reperfusion therapy.^4–9^ Thus, besides a precise estimation of the infarct core at admission, knowledge about the leptomeningeal collateralization is essential and may impact on clinical decision-making. Although different techniques have been proposed and are applied in the clinical setting for the assessment of collaterals, displaying these reliably remains a major challenge in stroke imaging.^10^

Due to wider availability and ease of use in acute stroke, computed tomography (CT) with CT perfusion (CTP) imaging is used more frequently than magnetic resonance imaging (MRI) in routine care. Relative cerebral blood flow (rCBF) maps derived from CTP provide a good approximation of the infarct core, with a reported sensitivity of 66–93% and a specificity of 72–87% for identifying the ischaemic core as depicted by gold standard diffusion-weighted imaging (DWI).^11–16^ CTP accuracy in predicting this core depends on the algorithm used to derive rCBF and on the threshold applied to perfusion maps.^11,12,14,15^ Consequently, the accuracy with which CTP predicts the core infarct is somewhat inferior to DWI.^16^

MRI-based assessment of leptomeningeal collateralization is challenging. Time-of-flight (TOF) MR angiography (MRA) is frequently used in clinical stroke imaging protocols^17^ to detect proximal large-vessel occlusion. To a degree, this technique allows for assessment of the collateral vessel abundance. However, the visualization of distal collaterals in proximal large artery occlusion can be impaired due to spin saturation at low flow conditions.^18–20^ Perfusion-weighted imaging (PWI) estimates the amount of tissue at risk. Conventional perfusion parameter maps provide information on leptomeningeal collaterals in proximal large artery occlusion.^21–25^ However, perfusion maps do not directly visualize collateralization. Furthermore, the calculation of these maps is model- and parameter-dependent and requires extensive image postprocessing leading to limited reliability and reproducibility.^26,27^

Various methods and scoring systems exist to directly assess collateral supply by the means of CT angiography (CTA)^28,29^ and digital subtraction angiography (DSA).^30,31^ However, the application of these techniques involves exposure to ionizing radiation, nephrotoxic contrast agents, and, in the case of DSA, invasive procedures.

If we were able to assess collateral vessels directly and reliably using MR PWI, collateral grading could be performed using existing stroke imaging protocols, avoiding the disadvantages of CTA and DSA in patients receiving stroke MRI at admission. In this proof-of-concept study, we hypothesized that collateral information can be extracted based on the magnitude of signal variance in dynamic susceptibility contrast (DSC)-based PWI. To assess the validity of a novel signal variance-based quantitative collateral index as an indicator of leptomeningeal collateral supply, we related this index to imaging-based parameters expected to reflect the degree of collateralization, and to clinical outcome measures. Furthermore, we compared the DSC-derived collateral index to established collateral scores derived from CTA and DSA.

## Materials and methods

### Study population

Selected from our database, patients admitted to our neurological department between April 2009 and October 2017 with acute proximal cerebral large vessel occlusion (ICA, MCA M1 or combined ICA/M1 occlusion) were included in the study if they had undergone an MRI investigation including DWI and PWI of sufficient quality to allow for coregistration and motion correction. This study was conducted according to the ethical standards on human experimentation of the local institutional review board (Goethe University Frankfurt, Faculty of Medicine), from which approval for the study was obtained. Written informed consent from patients was waived due to the retrospective character of the study.

### MRI protocol

MRI data were acquired as part of the clinical routine protocol on a Siemens 3 Tesla scanner (Magnetom Verio 3T; Siemens Healthcare, Erlangen, Germany), equipped with a body transmit and an eight-channel phased array head receive coil. Besides DWI to confirm acute cerebral ischaemia and PWI to estimate the amount of tissue at risk of infarction, the institutional stroke protocol included sequences for anatomical parenchymal and low flow imaging (conventional T2-weighted, FLAIR) plus sequences to detect intracerebral hemorrhage and assess thrombus length (T2*-weighted, susceptibility-weighted imaging). Furthermore, a three-dimensional TOF MRA was included for the detection of proximal large vessel occlusion and intracranial stenosis.

DWI data were acquired with a single-shot spin-echo echo-planar imaging (EPI) sequence, using the following parameters: echo time TE = 88 ms, repetition time TR = 4900 ms, field-of-view 220 × 220 mm^2^, matrix size 130 × 130, 25 axial slices, slice thickness 5 mm, inter-slice gap 0.5 mm and bandwidth BW = 1425 Hz/pixel. Diffusion sensitizing gradients were applied sequentially with b = 0, b = 500 s/mm^2^ and b = 1000 s/mm^2^. Apparent diffusion coefficient (ADC) maps were calculated using the commercially available scanner software.

Perfusion-weighted images were acquired with a DSC gradient-echo EPI sequence. Imaging parameters were TE = 30 ms, TR = 1500 ms, flip angle FA = 90 °, field-of-view 230 × 230 mm^2^, matrix size 128 × 128, 19 axial slices, slice thickness 4 mm, 1.2 mm inter-slice gap, BW = 1447 Hz/pixel, acquisition time 1:41 min. The intravenous contrast agent gadobutrol (0.1 mmol/kg; Gadovist® Bayer) was automatically applied by a power injector at a flow rate of 5 mL/s, followed by a bolus (20 mL) of 0.9% saline. The scanner software package was used to calculate non-deconvolved time-to-peak (TTP) maps.

### Theoretical considerations

In awareness of the potential technical limitations of this technique, the signal variance across time for each voxel in the PWI time series should be directly related to the delivery of contrast agent to the voxel as a measure of the presence of perfusion. Empirically, larger arterial blood vessels are characterized by a low mean signal intensity but a large bolus-related signal standard deviation across time in T2*-weighted PWI raw data, since the contrast agent causes a large and sharp signal drop in arteries, normally with a quick signal return to baseline. We therefore hypothesized that a way to isolate voxels reflecting blood vessels of the pial compartment in the PWI raw data might be to exploit the differences in relative signal variance (calculated as the standard deviation divided by the mean value) related to the application of intravenous contrast agent, whose magnitude can be expected to be higher in the larger feeding vessels of the pial compartment compared to the parenchyma and the perforating arteries.

### Image postprocessing and calculation of signal variance

The major part of the image postprocessing and analysis was performed automatically using an in-house built shell script, which used implemented tools provided in the FMRIB Software Library (FSL, http://www.fmrib.ox.ac.uk) toolbox for the image postprocessing and analysis steps described below. The information which hemisphere was affected in the individual patient was added as a prior to the script. Diffusion- and perfusion-weighted images were skull-stripped using ‘BET’^32^ prior to further postprocessing and analysis. To ensure sufficient anatomic contrast for coregistration, the first volume of the DWI time series (b = 0 s/mm^2^, purely T2-weighted) was extracted and linearly coregistered to the first volume of the PWI time series. Then, the resulting coregistration matrix was applied to coregister the ADC map, which was used for definition of the ischaemic core, to the perfusion-weighted images. PWI data were motion-corrected with ‘MCFLIRT’,^33^ using the mean volume across time as reference volume. Apart from motion correction, this tool was used to calculate maps depicting the standard deviation (σ) for every voxel in the PWI time series. Coefficient of variation (CV) maps were computed by applying the relationship

**Figure 1.**
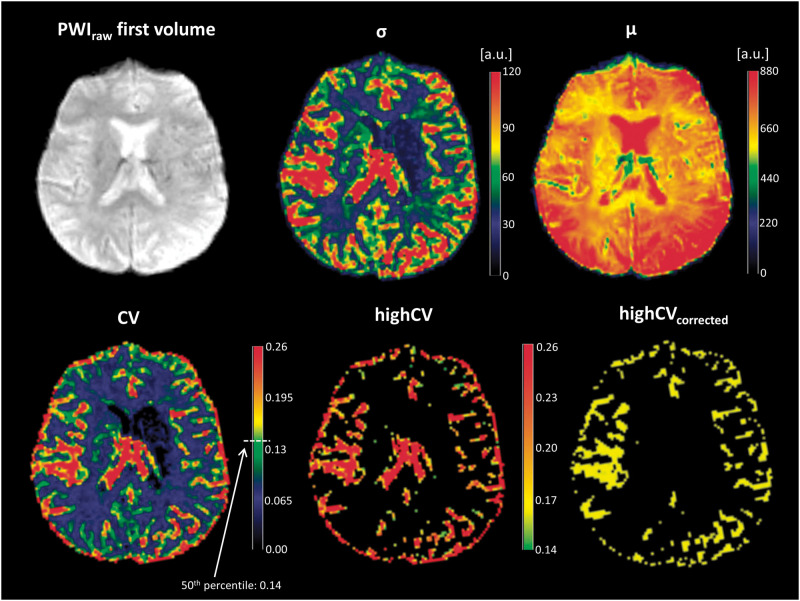
Illustration of calculation of signal variance in PWI. The top left image exemplarily shows the first volume of the PWI time series. Coefficient of variation (CV) maps were calculated by dividing the standard deviation of every voxel by its mean across time. Feeding vessels of the pial compartment are characterized by a large standard deviation and a relatively low mean signal intensity across time. CV maps (lower left image) were thresholded below the upper 50% of the robust range (lower middle image) and corrected for voxels representing the ventricles and outer CSF spaces. Colour bars represent robust intensity ranges. Standard deviations and mean values of signal intensity across time for the PWI raw data are given in arbitrary units, CV for each voxel is given as dimensionless number. Note the increased image contrast between larger vascular structures and brain parenchyma on the CV map (lower left image) compared to the standard deviation map (upper middle image). The corrected CV map is shown as a binary mask (lower right image). PWI: perfusion-weighted imaging; σ: standard deviation; µ: mean value; a.u.: arbitrary units; CV: coefficient of variance.

CV=σ/μ

where µ represents the mean of every voxel (Figure 1).

### Calculation of PWI-based collateral vessel index

As voxels with a highCV were expected to indicate the presence of blood vessels, maps labelled highCV were created by thresholding the CV maps, keeping only the upper 50% of the robust range for non-zero voxels (Figure 1). A ventricle mask derived from MNI152 standard space was used to exclude residual inner cerebrospinal fluid (CSF) spaces and choroid plexus from the highCV maps. Voxels representing outer CSF and cortical tissue adjacent to leptomeningeal vessels were found to show high signal variance at low overall signal intensity across time. To remove these voxels and minimize partial volume effects, the 5% of voxels with the lowest signal intensity at the time point of the maximum contrast-induced signal decrease were subtracted from the highCV maps (Figure 1). The calculation of the collateral vessel index (CVI_PWI_) is based on the assumption that the volume of leptomeningeal voxels with a highCV signifies the abundance of collateral vessels. To measure the respective highCV volume, a digital brain vessel atlas depicting the vessel density for each anatomic location^34^ was derived from MNI standard space and coregistered to the PWI data. After subtraction of the venous sinuses and large cerebral veins, the vessel density map was transformed into a binary mask and separated in the median plane. Finally, highCV volumes were extracted from the leptomeningeal compartment along the lateral and cranial convexity of each hemisphere and the CVI was calculated as

$$\begin{array}{c} \text{CV}{\text{I}}_{\text{PWI}}=\text{highC}{\text{V}}_{\text{affected}}\text{volume}(\text{m}{\text{m}}^{3})/\\ \text{highC}{\text{V}}_{\text{unaffected}}\text{volume}(\text{m}{\text{m}}^{3}) \end{array}$$

### Definition of the ischaemic core, PWI/DWI mismatch, tissue-at-risk and the hypoperfusion intensity ratio

To assess whether the newly developed collateral index presented here is suitable to depict collateral supply, we used different established imaging-based parameters that have been described and clinically used as predictors for a favourable clinical outcome and provide indirect information on leptomeningeal collateralization.

**Figure 2.**
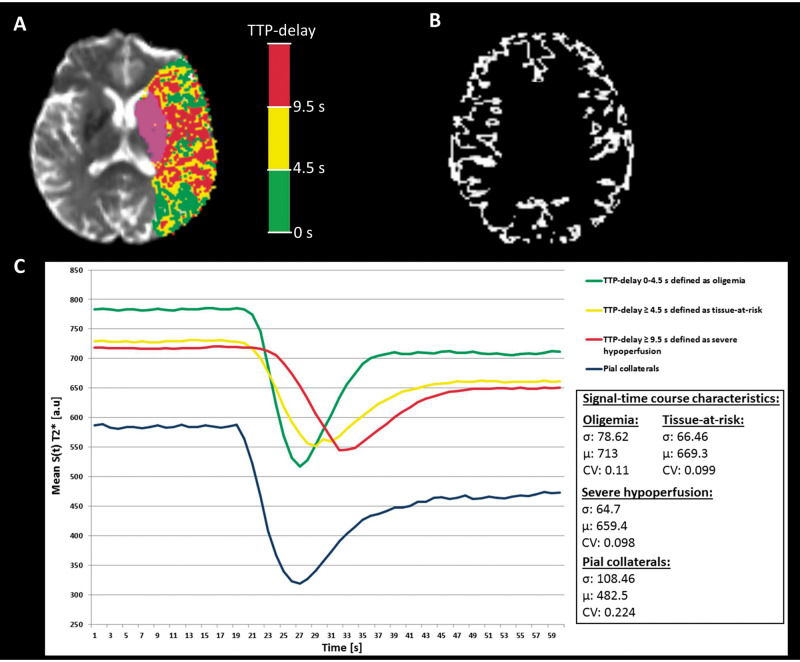
(a) Ischaemic core (violet) and different tissue compartments based on the severity of the TTP-delay overlaid on the first diffusion-weighted image (b = 0 s/mm^2^) of a representative patient (same patient as in Figure 1). The infarct core was defined on ADC maps applying a threshold of < 600 × 10^−6^ mm^2^/s. The scale bar shows the colour coding for different severities of TTP-delay defined as follows. Green: benign oligemia, yellow: tissue at risk, red: severely hypoperfused tissue. (b) Cortex mask used for measurement of the cortical infarct volume. (c) mean-signal time courses for each of the regions with TTP-delay shown in (a) and mean signal time course for adjacent pial collateral vessels. Major characteristics of the signal-time courses including standard deviation, mean signal intensity across time and coefficient of variation are provided in text form. It becomes clear that collateral vessels can be extracted from PWI raw data based on the magnitude of signal variance. TTP: time-to-peak; s: seconds; a.u.: arbitrary units; σ: standard deviation; µ: mean value; CV: coefficient of variation.

The ischaemic core at admission was defined using an established upper threshold of 600 × 10^−6^ mm^2^/s on ADC maps^21,35,36^ (Figure 2(a), violet region). Automatic segmentation using this ADC threshold provides a repeatable and objective estimate of the ischaemic core, as opposed to manual delineation of the infarct core.^35^ Previous research has shown that threshold-based segmentation of ADC maps reliably identifies the ischaemic core.^37^ When compared to trace images, ADC maps are less susceptible to certain artefacts (e.g. T2-shine-through, susceptibility pile-up, receiver coil-sensitivity-based intensity variation).^35,37^

Hypoperfused tissue in the territory of the occluded vessel was defined on the basis of TTP maps, applying the median time-to-peak of the contralesional hemisphere as lower threshold to generate maps of TTP-delay.^38^ The ischaemic core was subtracted from the entire TTP-delayed area to define the PWI/DWI mismatch region. The entire PWI/DWI mismatch ratio was calculated as the ratio between the entire perfusion lesion and the acute DWI lesion volume.

A minimum threshold of 4.5 s was applied to the TTP-delay map,^38–40^ which has been demonstrated to be equivalent to the more established threshold T_max_ ≥ 6 s,^38^ to define tissue at risk (Figure 2(a), yellow region). The PWI at risk/DWI mismatch ratio was calculated as the ratio between tissue at risk and the acute DWI lesion volume.

The hypoperfusion intensity ratio (HIR), which is presumed both to be associated with the degree of collateralization and to be a predictor of good functional outcome,^21^ was calculated as the proportion of the tissue at risk with a severe TTP-delay of ≥ 9.5 s (Figure 2(a), red region), equivalent to the ratio T_max_ ≥ 10 s/T_max_ ≥ 6 s as proposed by Olivot et al.^21,38^

The necessity of correcting for artefacts and voxels not representing brain tissue after automatic thresholding of ADC and TTP maps has been acknowledged in the literature.^35,38^ To this end, an automatic correction was implemented in the postprocessing algorithm. The functionality of this correction step is described in detail in the Supplemental Material. Automatically segmented and corrected ADC and TTP lesions were visually inspected by an experienced neuroradiologist and assessed for spatial dimension and plausibility. After the automatic correction procedure, no errors were observed.

Further, since we assumed cortical infarct volume to be a determinant of functional outcome and an indicator of the degree of pial collateralization in large vessel occlusion,^41^ the first diffusion-weighted image (b = 0 s/mm^2^) was segmented into grey and white matter using SPM12 (www.fil.ion.ucl.ac.uk/spm) implemented in MATLAB (http://www.mathworks.com). The tissue segmentation was conducted separately from the automatic processing described above. A cortex mask was created from the grey matter segmentation estimate and used to measure the cortical infarct volume (Figure 2(b)).

### Evaluation of leptomeningeal collateralization by DSA/CTA and assessment of final infarct volume

The combined analysis of DSA and CTA was used as gold standard for the evaluation of leptomeningeal collateralization. The PWI-based collateral score as proposed and introduced above was compared to the DSA/CTA results in terms of validity regarding infarct size and clinical outcome. For this purpose, available scoring systems for the setting of acute stroke were applied, which are based on DSA from the affected side and CTA in the arterial phase. For DSA, the collateral score developed by Christoforidis et al. was used for collateral grading. This score is based on the extent of angiographically visible retrograde filling of the MCA segments in the delayed venous phase.^30^ For CTA, we applied the scoring system developed by Menon et al., which uses the abundance of contrasted collateral vessels in different anatomic locations defined by the Alberta Stroke Program Early CT Score.^28^ Evaluation of leptomeningeal collateralization by CTA/DSA was performed by two experienced neuroradiological readers in consensus who were blinded to clinical and DSC-PWI data. In cases where both CTA and DSA were available, both modalities were used for collateral grading. Final infarct volume was determined on CT or MRI 2–5 days after symptom onset by manually tracing hypointense and hyperintense areas on CT and DWI, respectively. Whether MRI or CT was performed as follow-up imaging depended on time and logistical capacities in clinical routine and the clinical condition of the patient in each individual case.

### Statistical analysis

Testing for normal distribution was performed using the Kolmogorov–Smirnov test. Since several parameters were not normally distributed, we only applied non-parametric statistical testing. We compared medians between two groups using the Mann–Whitney U test. Correlations between collateralization and imaging parameters, between collateralization and clinical outcome measures, as well as between different collateral scores were assessed using Spearman's rank correlation. The CVI_PWI_ was dichotomized at the median to differentiate patients with good to moderate (CVI_PWI_≥0.963) and poor collaterals (CVI_PWI_<0.963). A receiver operating characteristic (ROC) curve analysis was performed to determine the power and optimal cutoff value for the CVI_PWI_ to discriminate patients with good and poor functional outcome. For the identification of independent predictors of favourable clinical outcome, we used a stepwise backward logistic regression model in which age, initial NIHSS, baseline DWI lesion volume, DWI/at risk mismatch ratio, HIR, the volume of severe hypoperfusion (TTP-delay ≥ 9.5 s), intravenous thrombolysis with recombinant tissue plasminogen activator (rt-PA), successful reperfusion and categorical CVI_PWI_ (cutoff value obtained from ROC curve analysis) were entered as variables. All tests were two-tailed and statistical significance was set to p<0.05. Statistical analysis was performed by the means of SPSS 22 (IBM, Armonk, NY).

## Results

### Baseline characteristics

Fifty-five patients (31 female, mean age 67.5 ± 15.7 years) met the inclusion criteria listed above. Median NIHSS at admission was 12, interquartile change (IQR) 8–16. Median time from symptom onset to MRI was 4.05 h (IQR 3–5.35). According to the TOAST criteria, stroke aetiology was classified as: (1) cardioembolic: 22 cases (40%), (2) due to large-artery atherosclerosis: 17 cases (30.9%), (3) due to rare causes including dissection and hypercoagulopathy: 4 cases (7.3%). Stroke aetiology remained unclear in 12 cases (21.8%). Twenty-nine patients (52.7%) received intravenous (i.v.) thrombolysis and 26 patients (47.3%) underwent endovascular thrombectomy. From the latter group, 21 patients (80.8%) had received bridging rt-PA before endovascular treatment. Twenty-one patients in the thrombectomy group had successful reperfusion with thrombolysis in cerebral infarction (TICI) score 2b or 3, while the other five patients showed no or incomplete reperfusion (TICI 0–2a). Median infarct volume at admission was 20.1 cm^3^ (IQR 7.8–38.6) and median ratios for the entire PWI/DWI mismatch and the PWI at risk/DWI mismatch were 10.99 (IQR 5.09–21.98) and 3.59 (IQR 2.12–8.41), respectively. Median HIR was 0.345 (IQR 0.247–0.513). Automatic image postprocessing, including all registration and correction procedures, calculation of lesion volumes and calculation of CVI_PWI_ required 02:25 minutes on a commercially available personal computer with a 3.4-GHz Intel Core i5 and 8 GB of RAM. CTA and DSA for collateral grading were available for 24 (43.6%) and 25 (45.5%) patients, respectively.

### Associations between CVI_PWI_ and other baseline imaging parameters

There was a significant negative correlation between CVI_PWI_ and ischaemic core volume at admission (rho = −0.459, p = 0.0001). After separation of cortical and subcortical infarct volume, the modulus of the negative correlation between CVI_PWI_ and cortical infarct volume (rho = −0.473, p = 0.0001) was slightly higher than the respective value for the correlation between CVI_PWI_ and subcortical infarct volume (rho = −0.402, p = 0.002). Significant positive correlations were found between CVI_PWI_ and the entire PWI/DWI mismatch ratio (rho = 0.494, p = 0.0001) and the PWI at risk/DWI mismatch ratio (rho = 0.400, p = 0.002), while the HIR correlated negatively with CVI_PWI_ (rho = −0.286, p = 0.035).

Patients with good to moderate collaterals (CVI_PWI_≥0.963) had significantly smaller baseline ischaemic core volumes and reduced cortical involvement (p<0.01) than patients with poor collaterals (CVI_PWI_<0.963). Furthermore, patients with CVI_PWI_ above the median had larger entire PWI/DWI and PWI at risk/DWI mismatch ratios (p<0.01) and smaller HIR (p<0.05) (Table 1).

**Table 1.** Univariate analysis comparing patients with good/moderate (CVI_PWI_≥0.963) and poor collaterals (CVI_PWI_<0.963).

	Good/moderate collaterals (CVI_PWI_≥0.963)	Poor collaterals (CVI_PWI_<0.963)	p-Value
No.	29	26	
Age (mean±SD)	67.5 ± 15.74	72.5 ± 15.3	0.879
Female (n (%))	16 (55.2)	15 (57.7)	1.000
NIHSS admission	11.5 (8–15.75)	14 (8.5–17.5)	0.091
Baseline infarct volume (cm^3^)	20.11 (7.84–38.59)	27.4 (16.02–65.16)	0.004
Baseline cortical infarct volume (cm^3^)	5.28 (1.45–13.75)	12.62 (2.64–21.43)	0.006
PWI/DWI mismatch ratio	10.99 (5.09–21.98)	6.7 (2.71–12.82)	0.001
PWI at risk/DWI mismatch ratio	3.59 (2.12–8.41)	2.4 (0.85–4.42)	0.003
Severe hypoperfusion (cm^3^)	29.08 (7.27–47.82)	31.56 (7.93–44.29)	0.607
HIR	0.34 (0.25–0.51)	0.47 (0.32–0.58)	0.024
Thrombolysis (n (%))	14 (48.3%)	15 (57.7%)	0.591
Reperfusion TICI 2b/3 (n (%))	12 (41.4%)	9 (34.6%)	0.782
Final infarct volume (cm^3^)	53.5 (13.25–168.75)	70.97 (41.28–169.58)	0.05
Infarct growth (cm^3^)	16.4 (3.41–58.45)	45.84 (13.58–130.73)	0.155
NIHSS discharge	5.5 (2–10.25)	8 (5–13)	0.133
NIHSS improvement	4 (2–8)	4.5 (0–8)	0.015
mRS discharge	3 (2–4)	4 (3–5)	0.081
Favourable outcome (n (%))	18 (62.1)	5 (23.8)	0.002

Note: Data are given as median (interquartile range). The Fisher exact test and the Mann–Whitney U test were used for categorical and continuous variables, respectively.

NIHSS: National Institute of Health Stroke Scale; DWI: diffusion-weighted imaging; PWI: perfusion-weighted imaging; cm^3^: cubic centimeters; HIR: hypoperfusion intensity ratio; TICI: thrombolysis in cerebral infarction score; mRS: modified Rankin Scale.

There were no significant correlations between CVI_PWI_ and collateral scores assessed with DSA and CTA (rho = −0.237, p = 0.255 and rho = 0.15, p = 0.496). Furthermore, DSA- and CTA-based collateral scores did not show a significant mutual correlation (rho = −0.128, p = 0.9). A detailed comparison of the results from collateral scoring for the modalities is given in Table 2.

**Table 2.** Comparison of the results from collateral scoring obtained with all three modalities.

CVI_PWI_	CTA (n = 24)	DSA (n = 25)
1.005	7	5
1.013	n.a.	3
0.87	n.a.	5
0.707	8	n.a.
0.86	13	n.a.
0.893	17	5
0.992	19	2
0.989	20	n.a.
0.953	n.a.	1
1.141	20	5
0.657	12	4
0.666	20	5
0.873	19	3
1.163	20	1
0.926	n.a.	1
1.18	n.a.	5
0.785	n.a.	4
1.113	20	n.a.
1.011	20	n.a.
0.694	14	4
0.953	19	5
1.249	n.a.	1
0.843	18	5
1.127	n.a.	3
0.975	n.a.	1
0.763	17	1
1.229	20	n.a.
0.958	11	5
1.129	2	n.a.
1.03	n.a.	3
0.997	9	n.a.
0.991	12	1
1.036	n.a.	1
1.246	10	n.a.

Note: The score for collateral assessment with CTA ranges from 0 (poor collaterals) to 20 (good collaterals), the score used for DSA from 1 (good collaterals) to 5 (poor collaterals).

CVI_PWI_: PWI-based collateral vessel index; CTA: computer tomography angiography; DSA: digital subtraction angiography; n.a.: not applicable.

### Associations between CVI_PWI_ and imaging-based and clinical outcome measures

In a pooled analysis of all patients included there was a strong negative correlation between both CVI_PWI_ and final infarct volume (rho = −0.430, p = 0.002) and CVI_PWI_ and absolute infarct growth (rho = −0.308, p = 0.031). Furthermore, CVI_PWI_ correlated negatively with NIHSS (rho = −0.341, p = 0.015) and modified Rankin Scale (mRS) at discharge (rho = −0.305, p = 0.023).

Final infarct volume was smaller in patients with good to moderate collaterals with a strong trend towards significance (p = 0.05). Furthermore, patients with good to moderate collaterals had a larger magnitude of NIHSS improvement until discharge (p<0.05) than patients with poor collaterals. Favourable outcome (mRS 0–2) was significantly more frequent in patients with good to moderate collaterals (p = 0.002) (Table 1). In order to exclude that these differences were mediated by successful reperfusion, we compared the rates of thrombolysis and successful endovascular treatment between the groups. No significant differences were found for thrombolysis (p = 0.591) and successful reperfusion TICI 2b/3 (p = 0.782) (Table 1). According to the results of the ROC curve analysis, there was a good discriminative power of CVI_PWI_ for predicting favourable clinical outcome (AUC: 0.71, p = 0.007). The cutoff value for CVI_PWI_ was 0.96 with a sensitivity of 78.3% and a specificity of 65.5%. In multivariate stepwise backward logistic regression analysis for good clinical outcome (mRS 0–2), the HIR (β = −4.11, odds ratio (OR) = 0.016, 95% confidence interval (CI) 0–2.38, p = 0.105), the categorical CVI_PWI_ (β = 2.8, OR = 16.39, 95% CI 1.42–188.7, p = 0.025) and the NIHSS at admission (β = −0.69, OR = 0.501, 95% CI 0.31–0.81, p = 0.005) remained in the analysis, showing significant results only for the categorical CVI_PWI_ and the NIHSS at admission. Consequently, these parameters were identified as independent predictors for good clinical outcome. Detailed results from the logistic regression analysis are given in Table 3.

**Table 3.** Stepwise multivariate logistic regression analysis for favourable clinical outcome (mRS≤2).

	β	Odds ratio	95% CI	p-Value
First step (multivariate r^2^ = 0.75)				
Age	0.088	1.092	0.99–1.2	0.067
NIHSS admission	−0.78	0.458	0.27–0.77	0.003
Baseline DWI volume (cm^3^)	0.009	1.009	0.97–1.05	0.665
PWI at risk/DWI mismatch ratio	−0.021	0.98	0.89–1.08	0.673
Severe hypoperfusion (cm^3^)	0.02	1.02	0.96–1.08	0.524
HIR	−7.19	0.001	0–6.21	0.118
rt-PA	0.16	1.71	0.17–8.34	0.874
Successful reperfusion (TICI score 2b/3)	0.99	2.7	0.29–25.22	0.385
CVI_PWI_ categorical (</ ≥ cutoff value)	2.34	10.36	0.93–114.89	0.057
Last step (multivariate r^2^ = 0.73)				
NIHSS admission	−0.69	0.501	0.31–0.81	0.005
HIR	−4.11	0.016	0–2.38	0.105
CVI_PWI_ categorical (</ ≥ cutoff value)	2.8	16.39	1.42–188.7	0.025

mRS: modified Rankin Scale; β: correlation coefficient; CI: confidence interval; NIHSS: National Institute of Health Stroke Scale; DWI: diffusion-weighted imaging; cm^3^: cubic centimeters; HIR: hypoperfusion intensity ratio; rt-PA: recombinant tissue plasminogen activator; TICI score: thrombolysis in cerebral infarction score; CVI_PWI_: PWI-based collateral vessel index.

Significant correlations with NIHSS and mRS at discharge were found neither for the DSA-based (rho = −0.048, p = 0.82 and rho = −0.129, p = 0.523) nor for the CTA-based collateral score (rho = −0.214, p = 0.365 and rho = −0.286, p = 0.196).

## Discussion

In this proof-of-concept study, a novel quantitative and rater-independent method for extracting collateral information from DSC perfusion data is presented. The findings of this study suggest that the signal variance-derived collateral index CVI_PWI_ provides information consistent with leptomeningeal collateralization in patients with proximal large artery occlusion, and that this method is superior to established CTA- and DSA-based methods for collateral scoring.

From the physiological point of view, collaterals are the main factor for maintaining tissue viability in case of an acute proximal cerebral vessel occlusion.^42^ The degree of collateralization has an impact on both the ischaemic core volume^43^ and pattern at the time of admission and patients with poor collaterals have larger cortical ischaemic lesions.^41^ Additionally, patients with a good collateral supply display a larger amount of salvageable tissue and less severe hypoperfusion^21,44^ and thus are more likely to be promising candidates for endovascular reperfusion.

**Figure 3.**
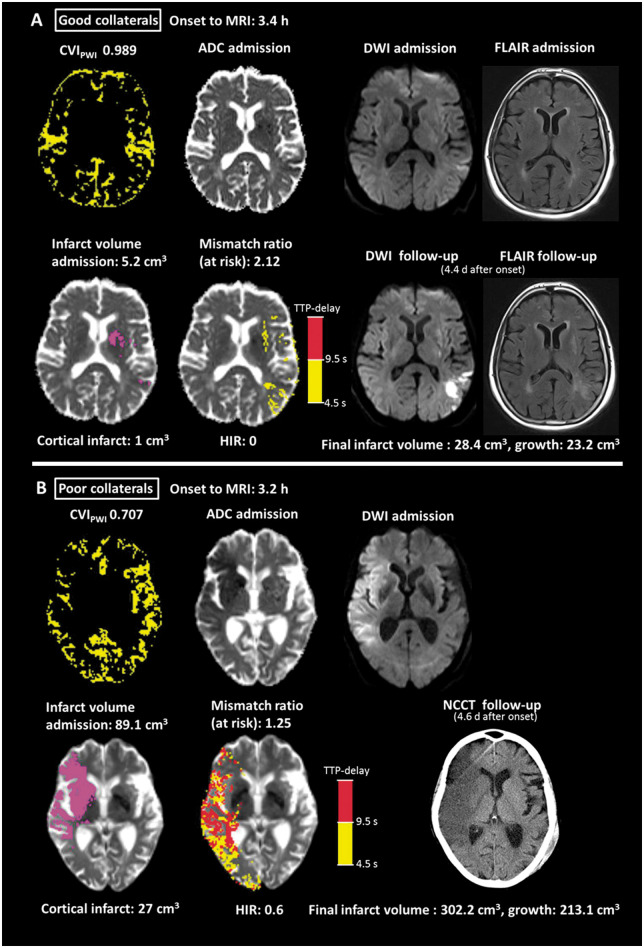
Images of two representative patients with good (a) and poor collaterals (b) with M1 occlusion and similar time from symptom onset to MRI (3.4 and 3.2 h). Both patients received intravenous thrombolysis but did not undergo endovascular treatment. (a) Female patient (73 y) with left M1 occlusion of unknown source. ADC map at admission shows small scattered infarcts in the left MCA territory (hypointense areas) involving the basal ganglia, parts of the insular ribbon, frontal operculum and the frontoparietal cortex. Some of these infarcts are not clearly visible in the b = 1000 s/mm^2^ diffusion-weighted image. FLAIR at admission shows hyperintense vessels over the left lateral convexity without clear demarcation of infarcts. Baseline ischaemic core defined by automatic thresholding and areas of TTP-delay are overlaid on the ADC map (lower left images). In this patient, no severe hypoperfusion (TTP-delay ≥ 9.5 s) is present, leading to a hypoperfusion intensity ratio of 0. Areas with a TTP-delay of ≥ 4.5 s outside the ischaemic core had progressed to infarction as visible on DWI and FLAIR at follow-up. NIHSS at discharge was 2 (improvement of four points compared to admission) and mRS was 1. (b) Female patient (81 y) with right M1 occlusion of cardioembolic origine. ADC and DWI maps at admission show extensive infarction involving large parts of the right MCA territory. FLAIR at admission was waived in this patient due to severe clinical conditions. Baseline ischaemic core defined by automatic thresholding and areas of TTP-delay are overlaid on the ADC map (lower left images). Areas with TTP-delay outside the ischaemic core showed progression to infarction at follow-up. NIHSS at discharge was 16 (deterioration of two points compared to admission) and mRS was 5. CVI_PWI_: PWI-based collateral vessel index; h: hours; ADC: apparent diffusion coefficient; DWI: diffusion-weighted imaging; FLAIR: fluid-attenuated inversion recovery; cm^3^: cubic centimeters; HIR: hypoperfusion intensity ratio; TTP: time-to-peak; s: seconds; NCCT: non-contrast computed tomography.

In this study, good leptomeningeal collateralization as indicated by CVI_PWI_ could be linked to smaller entire and cortical infarct volumes at baseline as well as to larger PWI/DWI mismatch ratios and less severe hypoperfusion as indicated by the HIR (Table 1, Figure 3). Interestingly, we did not find a significant correlation between CVI_PWI_ and the severity of the clinical deficit at admission (Table 1). This might indicate that good leptomeningeal collateral supply in patients with a complete proximal large-vessel occlusion does not attenuate the clinical deficit itself but rather preserves the mismatch between the ischaemic core and the clinical deficit, respectively, the still viable tissue at risk, for a longer period of time. Consistent with this assumption, it has been shown that the likeliness of a favourable outcome after endovascular therapy in patients with good leptomeningeal collateralization is less dependent on the time between symptom onset and reperfusion.^45^

In the clinical setting, collaterals as a neuroimaging parameter – as well as the ischaemic core volume – are an independent strong predictor of both response to reperfusion therapies and functional outcome.^7,46,47^ In our patient collective, the abundance of pial collateral vessels as depicted by the CVI_PWI_ was significantly inversely correlated with the final infarct volume and infarct growth at follow-up (Figure 3) as well as with the clinical deficit and functional outcome at discharge. This effect was independent of the administration of rt-PA and the status of reperfusion (Table 1). Furthermore, in multivariate logistic regression analysis the categorical CVI_PWI_ was an independent predictor of favourable functional outcome at discharge. The finding of the NIHSS at admission being a predictor of favourable functional outcome in multivariate regression might be explained by the heterogeneity concerning the application of endovascular treatment and successful reperfusion in this study. In summary, the results of this study are in line with the results of previous studies investigating the relationship between the direct surrogates of leptomeningeal collateral supply and clinical outcome measures.^8,46,47^

Numerous studies have addressed the possibility of obtaining direct or indirect information on collateral supply using PWI. Many of them have used conventionally applied perfusion measures like the time to maximum of the residue function (T_max_), rCBF and relative cerebral blood volume (rCBV),^21–25^ assuming that the severity of hypoperfusion in large artery occlusion is directly related to the abundance of collateral vessels. Although this assumption is plausible from a pathophysiological point of view and the results of these studies are promising, it should be noted that the calculation of these parameters – which requires the selection of an arterial input function – is subject to various sources of imprecision. Additionally, due to a lack of contrast in PWI parameter maps, the collateral vessel abundance cannot directly be assessed. Furthermore, the dependence of these parameters on the scan duration makes them susceptible to truncation-related errors.^48,49^ Consequently, as the approach used in this study relies on PWI source data and uses the signal characteristics induced by the contrast agent across time without additional image postprocessing it might produce more robust results. In addition, it allows for the direct delineation of collateral vessels and assessment of their volumetric abundance in the ischaemic hemisphere in comparison to the contralateral unaffected side. Since the approach in this study is based on the concept that the largest magnitude of signal variance across time can be found in the larger feeding vessels and thus does not consider the amount of contrast agent that is delivered to the tissue compartment via the perforating arteries, the reability of this method might be less limited by a shorter scan duration than the realibility of conventional established perfusion measures.^48,49^

In previous studies, apart from conventionally generated PWI parameter maps, PWI source data have also been applied to assess collateral flow in acute ischaemic stroke by using an image subtraction approach in order to visualize vascular filling at every time point in the PWI timeseries.^50–52^ Although this approach allows for the visualization of the temporal dynamics of collateral flow and precise assessment of collateral filling until the late venous phase, it carries the disadvantages of being operator-dependent and needs considerable time and effort in image postprocessing and analysis. In contrast, the signal variance-based approach proposed in this study requires minimal image postprocessing, is quantitative and rater-independent yielding the advantages of a single-shot technique. Another method for direct MRI-based assessment of collaterals is arterial spin labelling (ASL), which allows for visualization of collateral vessels due to its sensitivity to arterial arrival delays.^53–55^ Reliable calculation of CBF from ASL is still under investigation.^53^ However, ASL has been used for collateral assessment in chronic cerebral hypoperfusion^53,54^ and acute ischaemic stroke,^55,56^ providing promising results concerning the prediction of neurological outcome.^56^ The main advantages of this technique are the lack of contrast agent compared to DSC perfusion imaging.^55^ Technical limitations comprise the low signal-to-noise ratio compared to DSC and the susceptibility to patient motion,^55^ a particular problem in acute stroke. Furthermore, also CTP can be used to directly assess leptomeningeal collateralization in acute stroke, using a time-resolved approach.^57,58^ However, acquisition captures fewer images of the brain during bolus passage^57,58^ than in MR DSC perfusion, leading to limitations regarding late collateral filling. Furthermore, there are issues around radiation dose when repeatedly imaging the brain during bolus arrival. Currently, both methods are rater-dependent since they rely on visual rating scales.^57,58^

Conventional collateral scores obtained from CTA and DSA did not show significant correlations with CVI_PWI_ or clinical outcome measures at admission. This might be due to the fact that the scan duration of conventional CTA in some cases can be too short to detect collateral filling in the late venous phase and that the DSA-based collateral score used in this study is determined by unilateral angiographic examination of the affected side only and, therefore, might fail to capture the crossflow to leptomeningeal vessels from the contralateral unaffected side. The question whether PWI-based assessment of leptomeningeal collateralization has the potential to outperform CTA and DSA in certain aspects remains to be clarified. The overall processing time currently stands at 02:25 min (01:31 min for calculation of CVI_PWI_ only). However, employing on-line motion correction as available on the scanner, which would also provide mean signal intensities and standard deviation across time for the PWI time series, could substantially shorten this interval. By moving processing to the console, a delay of less than 45 s would be feasible. In conclusion, we believe that the automatic technique introduced in our work is feasible in the clinical setting of acute ischaemic stroke.

## Limitations

This study has several limitations. (1) Due to its retrospective character, the results of this study might be prone to unknown sources of bias. Therefore, they have to be interpreted with caution. (2) The patient collective is heterogeneous in terms of acute reperfusion therapies. Therefore, we cannot systematically assess the effect of leptomeningeal collateral supply on the response to reperfusion therapies. As the status of reperfusion was not explicitly evaluated in patients without endovascular treatment, non-documented spontaneous or rt-PA-related late reperfusion cannot be excluded in some patients. (3) The temporal dynamics of changes in collateral supply and potential collateral failure over time was not examined, which might have an additional impact on the clinical outcome, especially in patients who did not receive endovascular treatment. (4) Clinical outcome data of this study were available only for the time point of discharge, which might be different to the commonly used functional outcome at three months after stroke. (5) Arterial and venous vessels cannot reliably be distinguished for the method proposed. However, as venous outflow can be expected to be closely linked to the quantity of arterial inflow, this may not question the results of this study. A confirmation of the results of this study in a larger and more homogeneous patient collective using a prospective approach would be of interest.

## Conclusions

In summary, this study has shown the potential of quantitative and rater-independent assessment of leptomeningeal collateralization in acute stroke using a CVI derived from signal variance in PWI source data. Further research is required to confirm the validity of this method and to judge its applicability in the clinical setting and for clinical stroke trials.

## Supplemental Material

Supplemental material for Signal variance-based collateral index in DSC perfusion: A novel method to assess leptomeningeal collateralization in acute ischaemic stroke Click here for additional data file. Supplemental Material for Signal variance-based collateral index in DSC perfusion: A novel method to assess leptomeningeal collateralization in acute ischaemic stroke by Alexander Seiler, Arne Lauer, Ralf Deichmann, Ulrike Nöth, Eva Herrmann, Joachim Berkefeld, Oliver C Singer, Waltraud Pfeilschifter, Johannes C Klein and Marlies Wagner in Journal of Cerebral Blood Flow & Metabolism
