# Supplementary material for: Signal variance-based collateral index in DSC perfusion: A novel method to assess leptomeningeal collateralization in acute ischaemic stroke
Source: J Cereb Blood Flow Metab. 2019 Feb 13;40(3):574–87. doi: 10.1177/0271678X19831024 (PMC7025396; doi:10.1177/0271678X19831024)
Supplement: Supplemental material for Signal variance-based collateral index in DSC perfusion: A novel method to assess leptomeningeal collateralization in acute ischaemic stroke [file Supplemental3_Material.pdf]

Supplemental Material

Supplemental Methods

Description of correction of automatically segmented ischemic core and hypoperfusion lesions

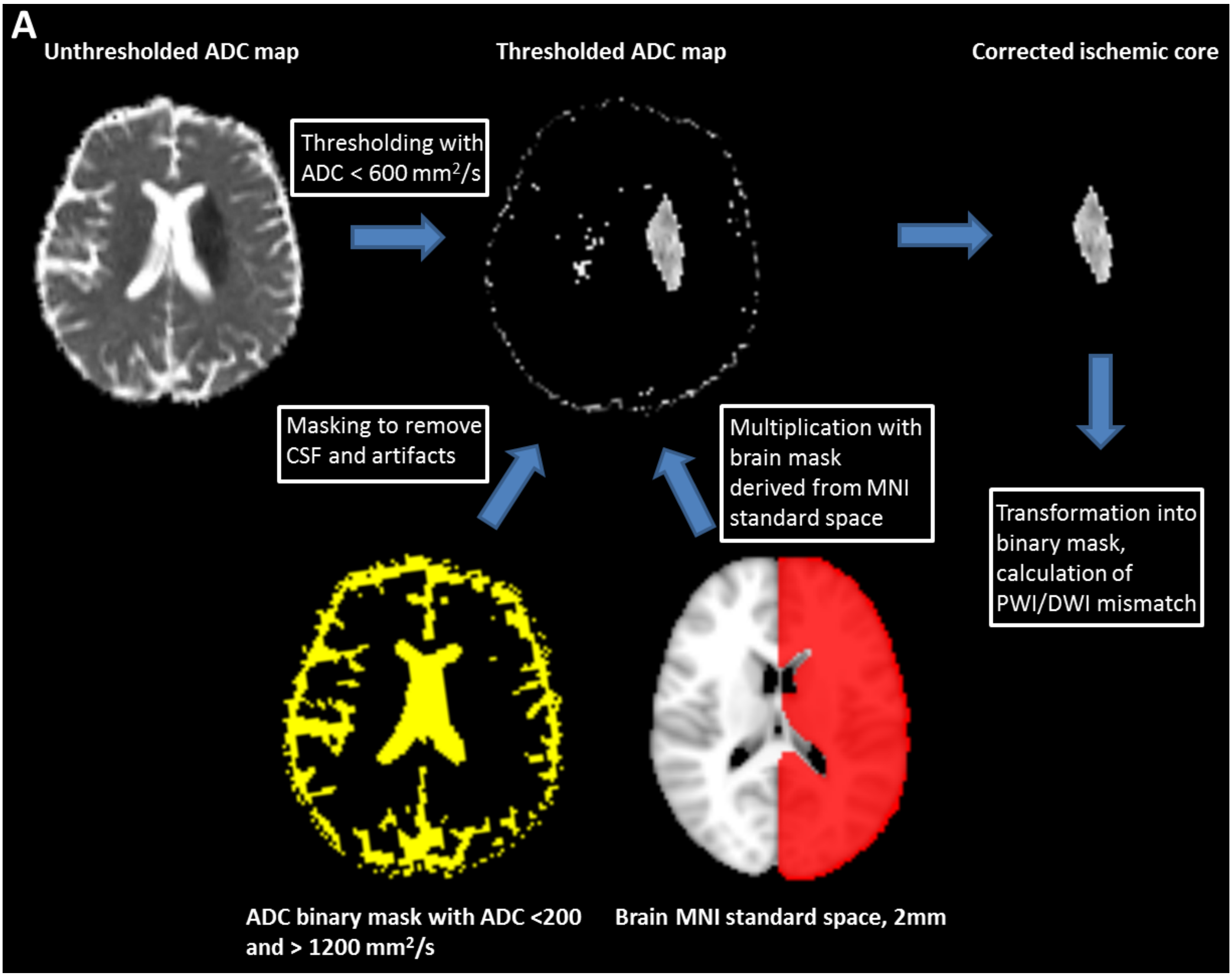

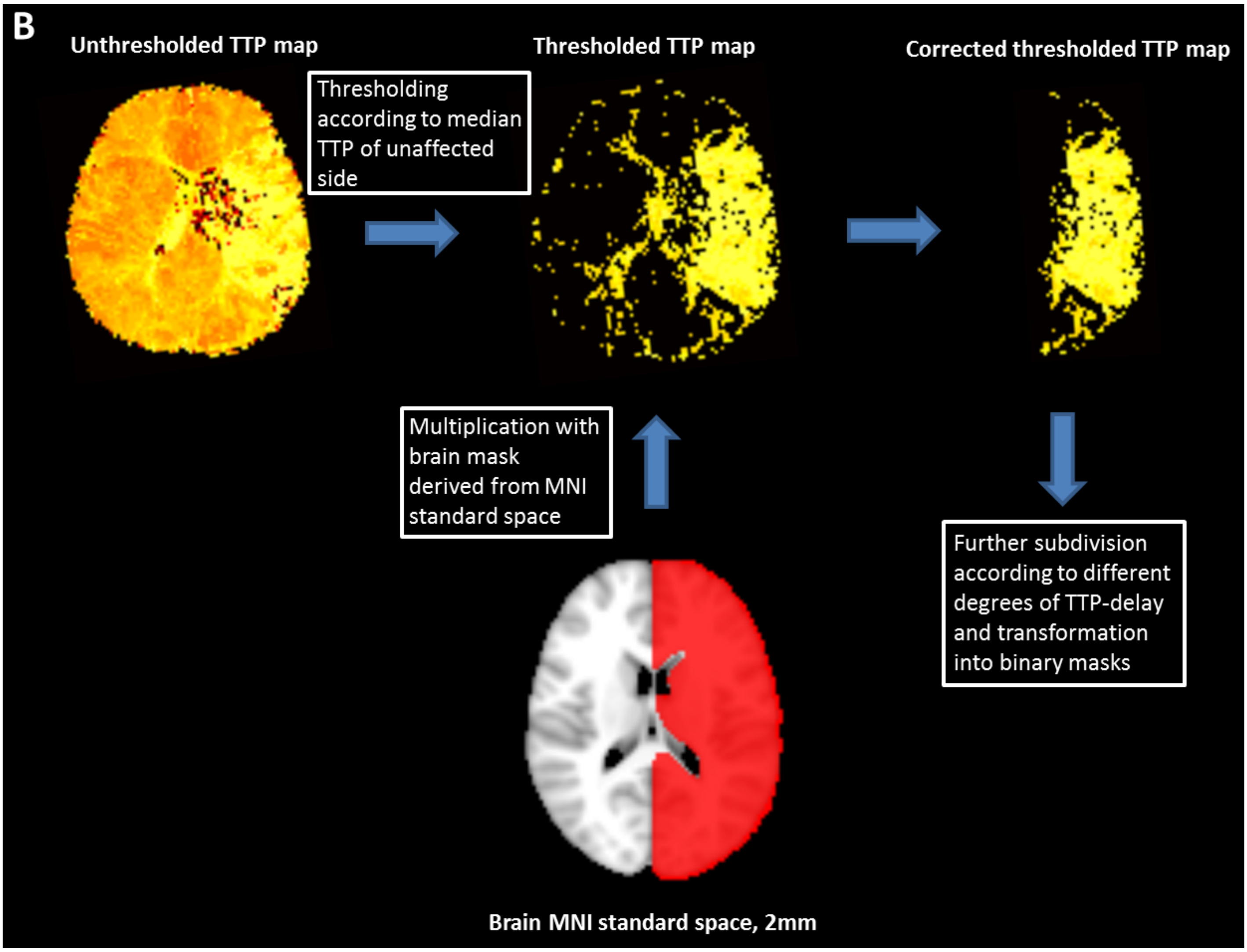

Supplemental Figure. A: Illustration of automatic correction of the segmented ischemic core lesions. After applying an automatic upper threshold of 600 x 10^{-6} mm^2/s to the ADC map, the thresholded ADC map still contains voxels representing inner and outer CSF as well as artifacts in the hemisphere contralateral to the ischemic lesion. These were removed by applying a binary mask containing only voxels with ADC values < 200 x 10^{-6} mm^2/s and > 1200 x 10^{-6} mm^2/s, which can be expected to represent CSF and undesired artifacts[1]. Furthermore, a brain mask of the affected hemisphere created in MNI standard space was applied to remove artifacts in the contralateral hemisphere. For transformation of the brain mask from MNI standard space to the diffusion-weighted image space, the first diffusion-weighted image (b=0 s/mm^2) was linearly coregistered to the MNI standard space. The resulting coregistration matrix was then inverted for the inverse transformation operation. During the inverse transformation, nearest neighbor interpolation was used to maintain the binary character of the mask. After this correction procedure, no errors are visible on the corrected image displaying the automatically segmented ischemic core. B: Illustration of automatic

correction of the segmented time-to-peak lesions. It becomes evident that after thresholding the TTP map according to the median time-to-peak from the unaffected hemisphere, the resulting thresholded TTP map has to be corrected for the lateral ventricles and artifacts in the hemisphere contralateral to the vessel occlusion. For this purpose, a brain mask of the affected hemisphere from MNI standard space excluding the lateral ventricles was applied to the thresholded TTP map. For transformation of the brain mask from MNI standard space to the perfusion-weighted image space, the first perfusion-weighted image was linearly coregistered to the MNI standard space. The resulting coregistration matrix was then inverted for the inverse transformation operation. During the inverse transformation, nearest neighbor interpolation was used to maintain the binary character of the mask. ADC: apparent diffusion coefficient; mm: millimeters; s: seconds; CSF: cerebrospinal fluid; MNI: Montreal Neurological Institute; PWI: perfusion-weighted imaging; DWI: diffusion-weighted imaging; TTP: time-to-peak.

Supplemental References:

1. Kidwell CS, Saver JL, Starkman S, et al. Late secondary ischemic injury in patients receiving intraarterial thrombolysis. *Ann Neurol*. 2002; 52: 698-703.
